# Chemokine regulation of inflammation during respiratory syncytial virus infection

**DOI:** 10.12688/f1000research.20061.1

**Published:** 2019-10-31

**Authors:** Rinat Nuriev, Cecilia Johansson

**Affiliations:** 1 National Heart and Lung Institute, Imperial College London, London, UK; 2 I. Mechnikov Research Institute for Vaccines and Sera, Moscow, Russian Federation

**Keywords:** RSV, chemokines, lung inflammation

## Abstract

Respiratory syncytial virus (RSV) can cause severe lower respiratory tract infections especially in infants, immunocompromised individuals and the elderly and is the most common cause of infant hospitalisation in the developed world. The immune responses against RSV are crucial for viral control and clearance but, if dysregulated, can also result in immunopathology and impaired gas exchange. Lung immunity to RSV and other respiratory viruses begins with the recruitment of immune cells from the bloodstream into the lungs. This inflammatory process is controlled largely by chemokines, which are small proteins that are produced in response to innate immune detection of the virus or the infection process. These chemokines serve as chemoattractants for granulocytes, monocytes, lymphocytes and other leukocytes. In this review, we highlight recent advances in the field of RSV infection and disease, focusing on how chemokines regulate virus-induced inflammation.

## Introduction

Respiratory syncytial virus (RSV) can cause upper and lower respiratory tract infections. Lower respiratory tract RSV infections are particularly common in young children, resulting in a spectrum of illnesses, including bronchiolitis and viral pneumonia ^1^. Infections caused by RSV occur worldwide, and it is estimated that over 3.2 million children under 5 years of age are hospitalised annually because of RSV infection ^2^. Moreover, RSV can cause lower respiratory tract infections in adults, especially in the elderly and immunocompromised, who are prone to more severe disease ^1, 3, 4^. Natural RSV infections result in incomplete immunity and therefore recurrent infections are common throughout life. The determinants of the outcome of RSV disease are not fully known, but both viral and host factors play a part ^5^. Among the latter are the immune responses elicited during RSV infection, which are crucial for efficient clearance of the virus but, if uncontrolled, can cause immunopathology. This can be detrimental for the lung tissues and result in impaired lung function and reduced oxygen exchange. Chemokines are crucial for the initiation of immune responses to RSV as they regulate leukocyte infiltration and localisation in the lungs ^6^. Alterations in the chemokine profile may therefore result in substantial dysregulation of immune responses. Insufficient or misdirected immunity may lead to increased viral replication and direct viral damage to the lung tissue. In contrast, unnecessarily hyperactive immune responses may have subsequent immunopathologic consequences.

## Innate immune responses during RSV infection

RSV infection often starts in the nasopharyngeal epithelium and rapidly spreads to the lower airways. The main cellular hosts for viral replication are the epithelial cells lining the airways and alveoli. When the virus reaches the lower airways, lung-resident cells such as epithelial cells, dendritic cells (DCs) and alveolar macrophages (AMs) initiate the innate immune response to the infection with the secretion of cytokines and chemokines ^1, 5^. AMs are crucial for the initial anti-viral responses as they are the main type I interferon (IFN) producers in the lung during RSV infection ^7^. Type I IFNs are cytokines that are important for inducing interferon-stimulated genes (ISGs) that limit viral replication and for priming and sustaining overall inflammatory cytokine and chemokine production ^8, 9^. The inflammatory chemokines orchestrate recruitment of blood leukocytes into the lung. *In vitro* studies show that epithelial cells and macrophages can produce chemokines (see details in Table 1). However, there is no clear evidence that AMs are the main source of most chemokines during RSV infection ^10, 11^ and many other cell types are likely involved in chemokine production. Interestingly, chemokine production is bi-phasic in mice ^12, 13^ and humans ^14^ after RSV infection; the first wave of chemokines is induced after sensing of the virus, and the second wave of chemokines is induced a few days after the initiation of infection. The second wave of chemokines correlates with the disease severity and the recruitment of T cells. The types of chemokines produced in the two waves are overall similar, but the underlying mechanism for the regulation and initiation of the two waves of chemokine production is not known. Therefore, increased knowledge of the regulation of chemokine production is important for the possibility to develop targeted therapies to reduce lung inflammation in the future.

**Table 1.** The most common chemokines produced during respiratory syncytial virus infection, their receptors, cell types they attract and possible sources.

Chemokine	Receptors	Cells attracted	Possible cellular sources	Study type	References
CXCL1 (KC)	CXCR1, CXCR2	Neutrophils	Stromal cells, neutrophils, ECs	Murine	^7, 8, 17, 19^
CXCL2 (MIP-2α)	CXCR2	Neutrophils	AMs?	Murine	^12, 19^
CXCL8 (IL-8)	CXCR1, CXCR2	Neutrophils	ECs, macrophages, neutrophils	Human	^14, 20– 28^
CXCL9 (MIG)	CXCR3	NK cells, T cells	?		^8, 33^
CX3CL1 (Fractalkine)	CX3CR1	Monocytes, NK cells, T cells	?	Murine	^34^
CXCL10 (IP-10)	CXCR3	Monocytes?, DCs, T cells	AMs, stromal cells?, ECs?	Human and murine	^8, 10, 14, 23,, 33, 35, 36^
CCL2 (MCP-1)	CCR2, CCR4	Monocytes, NK cells, eosinophils?	ECs?, macrophages?	Human and murine	^7, 10, 12, 22, 23, 25^
CCL3 (MIP-1α)	CCR1, CCR4, CCR5	Neutrophils, monocytes, NK cells, T cells	AMs, ECs, stromal cells	Human and murine	^8, 10, 13, 14, 19, 22, 23, 25, 30^
CCL5 (RANTES)	CCR1, CCR3, CCR5	Neutrophils, monocytes, DCs, NK cells, T cells	ECs, AMs	Human and murine	^11, 12, 14, 20, 31, 37^
CCL7 (MCP-3)	CCR2	Monocytes	?	Murine	^7^
CCL8 (MCP-2)	CCR1, CCR2, CCR3, CCR5	Monocytes, eosinophils, NK cells, T cells	?		
CCL11 (Eotaxin-1)	CCR2, CCR3, CCR5	Eosinophils, T cells	?	Murine	^19, 38, 39^
CCL12 (MCP-5)	CCR2	Monocytes, eosinophils, lymphocytes	Macrophages?	Murine	^7^
CCL17 (TARC)	CCR4	Th2 cells, Treg cells	?	Human	^40^
CCL20 (MIP-3a)	CCR6	DCs, T cells	?		
CCL22	CCR4	Th2 cells, Treg cells	DCs, macrophages		

AM, alveolar macrophage; DC, dendritic cell; EC, epithelial cell; NK, natural killer; Treg, regulatory T.

**Figure 1.**
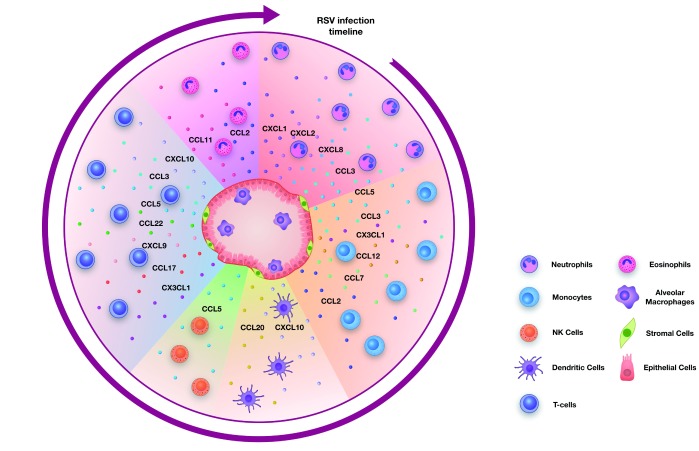
Chemokines as drivers of cell infiltration into the lung during respiratory syncytial virus (RSV) infection. Cells of the lung, such as alveolar macrophages, epithelial cells and stromal cells, produce chemokines during RSV infection to initiate and drive inflammation. During a primary RSV infection, neutrophils are the first cells to be recruited into the lung, followed by monocytes and dendritic cells. This is followed by the infiltration of natural killer (NK) cells and then T cells. During a secondary infection, tissue-resident and circulating memory T cells respond to the infection. In some cases, eosinophils can also infiltrate the lungs during RSV infection.

In this review, we describe the major chemoattractants (Table 1) considered to be important during RSV infection. We have summarised work from *in vivo* studies in mice and from human patient samples and describe the cell recruitment into the lungs after RSV infection based on timing, starting with the cell types infiltrating the lungs within hours of a primary infection and ending with the events occurring during secondary exposure, after re-encountering RSV ( Figure 1).

## Neutrophils during RSV infection

Neutrophils are the first cell type to arrive at a site of infection or tissue damage and they infiltrate the lung in both mice and humans in large numbers during RSV infection ^8, 15– 17^. Neutrophils are attracted into the lung tissue by a wide range of different molecules. These include not only several chemokines but also cytokines, eicosanoids and small peptides ^18^. In this review, only the chemokines will be discussed. CXCR2 and CCR1 are the most abundantly expressed chemokine receptors on neutrophils. CXCR2 is able to interact with a number of different chemokines, but CXCL1, CXCL2 and CXCL8 have been studied the most. Similarly, CCR1 can bind several distinct chemokines such as CCL3 and CCL5 ^18^.

CXCL1 (KC) and CXCL2 are considered to be some of the earliest chemokines expressed in the lungs of mice after RSV infection, detectable as early as 4 to 8 hours after virus exposure ^7, 8, 17, 19^. Moreover, recombinant CXCL1 can recruit large numbers of neutrophils into the lungs if given intranasally to mice ^17^. CXCL1 has been suggested to be produced by several different cell types, including epithelial cells ^20^ but not AMs ^10^. Recently, it was shown that a stromal cell type—that is, a non-epithelial (AT-II) and non-endothelial cell—is the main source of CXCL1 during RSV infection of mice ^17^.

CXCL8 (IL-8) has no orthologue in mice and can be studied in humans only. Many studies have found elevated CXCL8 levels in bronchoalveolar (BAL) fluid or nasal washes from RSV-infected children (for example, [ 20– 26]) and from RSV-challenged healthy adult volunteers ^14^. The origin of CXCL8 during RSV infection is not clear, but an *in vitro* model showed that primary paediatric bronchial epithelial cells can produce CXCL8 after RSV infection ^27^. Furthermore, RSV can directly trigger the release of CXCL8 from neutrophils ^28^. A recent study revealed links between viral load, CXCL8 levels and changes in the microbiome during RSV infection ^29^. In that study, the abundance of bacteria of the *Haemophilus* genus in nasopharyngeal aspirates of RSV-infected hospitalised infants was a predictor for CXCL8 levels and higher viral load ^29^.

CCL3 can recruit many different cell types such as neutrophils, monocytes, natural killer (NK) cells and T cells. CCL3 production in the lungs increases soon after RSV infection in mice ^8, 13, 19, 30^ and in infants ^22, 23, 25^. Although CCL3 can be produced by AMs ^10^, two studies using AM-depleted mice show different results: one shows a reduction in CCL3 ^31^ and the other no difference ^11^ when AMs are depleted during RSV infection. In addition, after RSV infection of BALB/c mice, CCL3 was detected in alveolar epithelial cells and endothelial cells ^32^. This suggests that CCL3 can originate from several cell types in the lung.

Neutrophils phagocytose microbes and release granules containing oxygen radicals, elastases and proteolytic enzymes ^41– 43^. In addition, they form neutrophil extracellular traps (NETs), which serve to stop pathogens from propagating ^44, 45^. Although the role of neutrophils is well defined during bacterial or fungal infections, their role during RSV infection remains unclear. It is not yet known whether they have a beneficial role limiting the spread of the virus or a detrimental role damaging the lung tissue ^46, 47^. The viral load does not change if neutrophils are depleted during RSV infection ^48^, suggesting that neutrophils do not have a substantial direct anti-viral role. However, the inflammatory environment in the lung, induced by RSV infection, results in neutrophil activation ^17^, and *in vitro* studies suggest that activated neutrophils augment the detachment of epithelium infected with RSV ^49, 50^. Furthermore, a detrimental role of excessive neutrophilic response is suggested by the fact that the degree of neutrophilic infiltration into the lungs correlates positively with severity of RSV-induced bronchiolitis ^15, 16, 51^. Also, infants with RSV-induced bronchiolitis have increased levels of neutrophil elastase ^21, 52^ and signs of oxidative burst ^53^, which can promote oxidative stress and tissue injury ^54^. NETs can be secreted by neutrophils from RSV-infected children and have also been detected in lungs of RSV-infected calves ^55^. *In vitro* studies have shown that ** RSV fusion protein can interact with TLR4, an innate immune receptor expressed on neutrophils and other cells, to trigger formation of NETs ^56^. It has also been suggested that NETs can capture RSV and that NET formation can contribute to lung damage during RSV infection ^55^. In sum, neutrophils are a key population of cells recruited into the lungs after RSV infection, but more studies are needed to confirm whether they are beneficial or detrimental to the host during RSV infection.

## Monocytes during RSV infection

Monocytes are the second cell type to infiltrate the lung after RSV infection. Human and murine monocytes are divided into two main subsets on the basis of their chemokine receptor expression. Their functions seem to be more or less similar, but one subset expresses high levels of CCR2 and low levels of CX3CR1 (CCR2 ^hi^ subset) and the other subset expresses high levels of CX3CR1 and low levels of CCR2 (CX3CR1 ^hi^ subset) ^57^. CX3CR1 is also expressed on T cells and airway epithelial cells ^58^. CX3CR1 binds to its ligand, CX3CL1, which is important for the chemotaxis of CX3CR1 ^hi^ monocytes as well as T cells. Furthermore, CX3CR1 expression on monocytes is important for their survival ^59^. During RSV infection of mice, CX3CR1 deficiency is associated with reduced innate immune cell recruitment, notably a significant decrease in NK cells and CD11b ^+^ cells (which may represent a monocytic subpopulation) ^34^. Interestingly, RSV G protein can bind directly to CX3CR1 and influence chemotaxis of lymphocytes ^60^, and CX3CR1 has been suggested to be a receptor used by RSV to infect cells ^1, 58^.

CCR2 is also an important receptor expressed on monocytes. CCR2 binds to CCL2, CCL7, CCL8 and CCL12, and the first two chemokines are generally considered to be the most important for monocyte recruitment ^57, 61^. However, both human and murine monocytes express CCR1 and CCR5, which means that they can also be recruited by the chemokines CCL3 and CCL5 ^57^. CCL2, CCL3, CCL5 and CX3CL1 have all been found in nasal samples or lung tissues of human and mice infected with RSV (for example, [ 7, 12, 14, 22, 23, 25]).

In mouse models, CCL2, CCL7 and CCL12 are produced early after RSV infection ^7, 12^. In humans, CCL2 levels correlate positively with disease severity: infants with RSV bronchiolitis who required mechanical ventilation show significantly elevated levels of CCL2 in BAL fluid compared with control infants intubated for non-infective causes ^22, 23^, and children with severe RSV disease displayed higher levels of CCL2 in nasopharyngeal wash samples than controls ^25^. The source of CCL2 during pulmonary inflammation has been under investigation but remains controversial. Experiments in *Mavs ^−/−^* and *Ifnar1 ^−/−^* mice show that CCL2 expression is promoted by type I IFNs produced by AMs ^7^. *In vitro* studies show that CCL2 can be produced by murine airway epithelial cells but not by AMs ^10, 12^. However, it is still unclear whether AMs can produce CCL2 *in vivo* or whether they simply promote chemokine expression by producing type I IFNs.

CCL5 (RANTES), another monocyte chemoattractant, is also considered to be important during initial responses to RSV infection. This chemokine binds to a wide range of receptors, including CCR1, CCR3 and CCR5, expressed on different types of immune cells: Th1 T cells, macrophages, DCs, neutrophils and NK cells ^6^. Moreover, it has been proposed that CCL5 has a direct anti-viral effect against RSV by blocking RSV fusion protein interactions with epithelial cells ^62^. Surprisingly, recent studies show that the levels of CCL5 are higher in nasal fluid samples of children with moderate RSV bronchiolitis compared with children with severe disease ^37^. AMs play a role in CCL5 production during RSV infection as AM depletion in mice results in decreased levels of CCL5 ^11, 31^. It is possible that AMs do not produce CCL5 themselves but exert their effects through the production of mediators such as type I IFNs that subsequently act on other cells to increase CCL5 production. Furthermore, *in vitro* studies show that human cord blood–derived mast cells ^63^ and human airway and bronchial epithelial cells can produce CCL5 and that CCL5 release depends on live virus ^12, 20^.

Monocyte-derived cells consist of inflammatory monocytes and monocyte-derived DCs and can constitute up to 40% of total lung leukocytes in the mouse model of RSV infection ^7^. Furthermore, monocyte-derived cells play a direct role in limiting RSV replication ^7^. Monocytes exhibit their anti-bacterial effects through the production of tumour necrosis factor (TNF) and inducible nitric oxide synthase ^64^, but how they limit RSV replication is not yet understood. Contrary to their anti-viral activities, monocytes can also have harmful effects on lung tissue. In an influenza virus– *Streptococcus pneumoniae* co-infection mouse model, inflammatory monocytes induced damage to the lung barrier by killing epithelial cells via a TNF-related apoptosis-inducing ligand (TRAIL)-dependent mechanism, resulting in decreased control of the infection and reduced animal survival ^65^. However, there are no studies revealing a harmful role of monocytes during RSV infection. Given that viral–bacterial and viral–viral co-infections can occur in immunocompromised children or when several viruses such as RSV, rhinovirus and influenza virus co-circulate at the same time ^66– 69^, it would be very interesting to investigate the exact role of monocytes during RSV infections.

## Dendritic cells during RSV infection

DCs are the main antigen-presenting cells that initiate the adaptive immune responses to infections. This function makes DCs especially important for the clearance of viral infections such as RSV. DCs are resident in the lung during homeostasis and can respond to RSV immediately. However, immature DCs (not clear whether these are monocyte-derived) can also be recruited to sites of inflammation by many inflammatory chemokines binding to CXCR1, CXCR3, CCR1, CCR2, CCR5 and CCR6 ^6, 70, 71^, and DCs are recruited to the nasal tissue in children with RSV infection ^72^. One chemokine associated with DC recruitment during RSV infection is CXCL10 as antibody-mediated neutralisation of CXCL10 results in impaired DC recruitment and maturation with reduced levels of type I IFN and IL-12p70 in the lungs of RSV-infected mice ^35^. Similar responses were observed after neutralisation of CXCR3, the only known receptor for CXCL10 ^35^. Additionally, RSV-infected mice treated with neutralising antibodies against CCL20 or CCR6 ^−/−^ mice, another DC chemoattractant and chemokine receptor respectively, recruit fewer conventional DCs but show reduced lung pathology ^36^. These data suggest that DCs can have both a beneficial and detrimental role in the lungs.

## Innate lymphoid cells during RSV infection

NK cells, part of the innate lymphoid cell 1 (ILC1) group, are important anti-viral innate lymphoid cells that activate other immune cells or kill virus-infected cells. NK cells, like other immune cells, express an extensive variety of chemokine receptors and can be attracted to the sites of inflammation via several distinct pathways ^6, 73^. The CCR5/CCL5 axis plays an important role in the accumulation of NK cells at virally infected sites, and during influenza virus infection, both CXCR3 and CCR5 have been shown to be important for NK cell recruitment ^74^.

NK cells are recruited to the lungs of RSV-infected mice and get activated to produce IFN-γ ^75^. *Ex vivo*, human NK cells can be infected by RSV, especially in the presence of RSV-specific antibodies ^76^. However, the number of human NK cells has also been shown to decrease with severe RSV disease ^77^, and if NK cells are depleted from mice, IFN-γ production is suppressed and more of a Th2 response develops ^78^.

RSV has also been shown to activate IL-13–producing ILC2s via the production of TSLP ^79^, and STAT-1 signalling was shown to be important for the activation of ILC1s and the repression of ILC2s and ILC3s ^80^. Overall, very little is known of the recruitment of ILCs during RSV infection, and more information will aid in the understanding of how they are recruited and their contribution to viral clearance or lung damage.

## Adaptive immune responses during RSV infection

Cells of the adaptive immune response infiltrate the lung both during primary and secondary infections. These are mostly T cells: CD8 ^+^ (CTL) T cells and CD4 ^+^ T cells (both T helper cells and regulatory T [Treg] cells) ^1^. After naïve T cells have been primed in lymph nodes, they migrate to the lungs in response to chemotactic signals. In mice, it is known that RSV infections lead to increased numbers of T cells in the lung tissue, which typically peak at day 7 or 8 following a primary infection ^81, 82^. T cells accumulate at a similar time (8 to 10 days after infection) in the human airways after RSV infection of healthy volunteers ^83^. Interestingly, the final lung viral clearance, both in mice and humans, occurs on days 8 to 10 after RSV infection, corresponding to the peak of adaptive immune responses ^1, 83, 84^.

Chemokines, such as CCL3, CXCL9 and CXCL10, regulate the infiltration of effector T cells into the lungs and they are all produced during RSV infection in mice and humans ^8, 13, 19, 23, 82, 33^. Memory T cells are formed after the first encounter with RSV. These are both effector memory cells and lung-resident memory T cells (Trm cells). The Trm cells provide a quick response during subsequent infections ^85^, whereas the effector memory cells need to be recruited upon re-infection ^1, 84^. Chemokine signalling is therefore considered to be an important regulatory mechanism in the formation of, especially, long-term memory CD8 ^+^ T-cell populations in the lung ^86^. It has been observed that, following influenza virus infection, mice deficient in either CXCR3 or CCR5 have significantly elevated numbers of memory CD8 ^+^ T cells. Although it is not completely clear for RSV infections, these data suggest that chemokine signalling through CXCR3 and CCR5 can regulate the effector versus memory T cell recruitment into the lung ^86^.

Interestingly, CCL17 and CCL22 can recruit both Th2 cells and Treg cells into the lungs ^87^. CCL17 recruits Th2 cells, especially in mice sensitised by vaccinia virus expressing the RSV G protein before RSV infection ^88^, and serum CCL17 is increased in RSV-infected children compared with children with other respiratory infections or healthy controls ^40^. Furthermore, RSV-specific CD8 ^+^ T cells present in the lung can inhibit the production of CCL17 and CCL22 and therefore limit the recruitment of Th2 cells ^89^.

Thus, chemokines are important during both primary and secondary RSV infection as they regulate effector, memory T and Treg cell recruitment and thereby can determine the extent of disease severity during RSV infection. More detailed studies of how the chemokines also determine the exact localisation of effector, Treg and memory T cells and thereby direct their effector functions will be important for future work.

## Eosinophils during RSV infection

Generally, eosinophils are not considered to have an important role during primary viral infections. However, during memory responses to RSV infection, eosinophils can infiltrate the lungs. This was especially the case when children, or mice, were vaccinated with formalin-inactivated RSV (FI-RSV). This vaccination induced a Th2-biased memory response with Th2 cells and pulmonary eosinophilia following RSV challenge, resulting in increased disease severity ^1, 90– 92^. For a long time, it was believed that lung eosinophilia was the driving factor of the FI-RSV vaccine-enhanced disease. However, more recent studies and re-evaluation of the initial vaccine trials revealed that eosinophilic infiltration was not the only characteristic component of vaccine-enhanced disease, suggesting that other factors may be important ^91, 93^.

Eosinophils can be attracted to the lungs by chemokines such as CCL2 or CCL11. CCL2 and its role in chemotaxis of monocytes were extensively discussed above. ** CCL11, also called eotaxin, is considered to be the main chemokine for eosinophil recruitment. Mice sensitised by vaccinia virus expressing the RSV G protein showed eosinophils in the lungs following subsequent RSV infection but after administration of anti-CCL11 antibodies showed significantly reduced lung eosinophil numbers. Moreover, CCL11 depletion resulted in subsequent decrease in CD4 ^+^ T-cell influx to the lungs and decreased IL-5 production with no influence on the viral load ^19, 38^. However, more recent studies of vaccine-enhanced RSV disease suggest that eosinophils are pro-inflammatory and have direct anti-viral functions during RSV infection. Experiments in eotaxin knockout mice show complete absence of eosinophils in the lungs of FI-RSV immunised mice following RSV infection with reduced lung inflammation. However, the eotaxin knockout mice had significantly higher lung RSV titres compared with wild-type mice, and when lung eosinophilia was restored, by either intratracheal rCCL11 administration or adoptive transfer of eosinophils, this resulted in increased viral clearance ^39^. These data raise the question again, do eosinophils have a positive or negative influence on the course of RSV infection?

## Conclusions

Chemokines are key drivers of the anti-viral inflammatory response during RSV infection. Many chemokines are produced during the infection, and specific cell types are recruited via several unique chemokine/chemokine receptor interactions. The redundancy of chemokines in cell recruitment denotes the importance for the host of being able to attract immune cells into the lungs to help combat the infection. We still know very little about the cellular sources of chemokines in the lung, and in order to identify the main cellular source (or sources) of a chemokine during the course of infection, several lung cell types have to be compared side by side which can be performed only *in vivo* or from biopsies. Also, how chemokines direct the migration of immune cells within the lung tissue to determine their precise localisation, which will have implications for their effector functions, is an important future research avenue.

Almost all chemokines correlate positively with disease severity during RSV infection ^25, 26^. This observation is most likely explained by the scenario that excessive inflammatory responses in the delicate lung tissue will drive immunopathology via cell activation and mediator release. We are still far from being able to use chemokine receptor blockade as a treatment for RSV-induced disease (as discussed in more detail in 94). However, greater in-depth knowledge of which cell types act as the main sources of chemokines and how chemokine production is regulated will help the understanding of the initiation and maintenance of inflammation in the lung and possibly a more targeted approach for reducing lung inflammation via chemokine/chemokine receptor inhibition in the future.
